# Biological treatment of pesticide-containing wastewater from coffee crops: selection and optimization of a biomixture and biobed design

**DOI:** 10.3389/fmicb.2024.1357839

**Published:** 2024-02-06

**Authors:** Fernando Oviedo-Matamoros, Marta E. Pérez-Villanueva, Mario Masís-Mora, Rónald Aguilar-Álvarez, Didier Ramírez-Morales, Michael Méndez-Rivera, Carlos E. Rodríguez-Rodríguez

**Affiliations:** ^1^Centro de Investigación en Contaminación Ambiental (CICA), Universidad de Costa Rica, San José, Costa Rica; ^2^School of Biosystems Engineering, Universidad de Costa Rica, San José, Costa Rica

**Keywords:** coffee, pesticide, toxicity, removal, optimization, wastewater

## Abstract

The biopurification systems (BPS) or biobeds are employed for the treatment of pesticide-containing wastewater of agricultural origin. The use of these devices for pesticide removal requires the proper optimization of the composition of biomixtures (BPS active matrix) according to the target pesticides applied on a specific crop and the available materials used in their elaboration. This work aims to design a biomixture for the simultaneous treatment of several pesticides applied in coffee crops, according to local practices in Costa Rica. Three biomixtures containing either coffee husk, coconut fiber or rice husk (as the lignocellulosic substrate) were applied for the removal of 12 pesticides. The profiles of pesticide elimination and the mineralization of radiolabeled chlorpyrifos (^14^C-chlorpyrifos) revealed that the best performance was achieved with the coconut fiber biomixture, even though similar detoxification patterns were determined in every biomixture (according to immobilization in *Daphnia magna* and germination tests in *Lactuca sativa*). The optimization of this biomixture’s composition by means of a central composite design permitted the definition of two optimal compositions (compost:soil:coconut fiber, % v/v) that maximized pesticide removal: i. 29:7.3:63.7 and ii. 11:7.3:81.7. The validation of these optimized compositions also included the use of an alternative soil from another coffee farm and resulted in overall DT_50_ values of 7.8–9.0 d for the pesticide mixture. Considering the removal kinetics in the optimized biomixture, a 1 m^3^ BPS prototype was dimensioned to be eventually used in local coffee farms. This work provides relevant information for the design and implementation of BPS at on-farm conditions for the treatment of pesticide-containing wastewater of a major crop.

## 1 Introduction

Coffee production represents one of the major agricultural activities in Costa Rica, aiming both to local and international markets. By 2022 there were 77352.49 Ha of sown fields and a production of 436473.3 metric tons, according to the National Agricultural Survey 2022 (INEC, 2022). As with most crops, coffee production requires the use of diverse agrochemicals, including several pesticides; nowadays, more than 45 organic pesticides from different chemical groups are registered for application on coffee fields in Costa Rica [Servicio Fitosanitario del Estado (SFE), 2023].

The point-source contamination with pesticides has been referred to as the main cause of pollution of water streams at farm level (Castillo et al., 2008). Such point-sources include leakages during the filling of spraying equipment and incorrect disposal of residues produced during the washing of machinery after pesticide application or the rinsing of pesticide formulation containers (Castillo et al., 2008; Dias et al., 2020; Rodríguez-Rodríguez et al., 2021). Therefore, the proper handling, disposal and treatment of such pesticide-containing wastewater represents a key step in farm management to prevent the exposure of the surrounding terrestrial and aquatic environments.

Biopurification systems (BPS) or biobeds comprise an eco-friendly biotechnological approach for the disposal and treatment of pesticide-containing wastewater of agricultural origin. The removal of pesticides in such devices is expected to be faster than in the environment (i.e., soil), largely due to the biodegradation that takes place in the biomixture, which is the biologically active matrix in the BPS (Karanasios et al., 2012). The biomixture is made up of three components, namely, a lignocellulosic substrate (used for the colonization and activity of ligninolytic fungi of demonstrated capacity to transform organic pollutants such as pesticides; Xiao et al., 2011; Rodríguez-Rodríguez et al., 2013), a humic-rich matrix (commonly peat or compost, to prompt the adsorption of pesticides; Castillo et al., 2008) and soil pre-exposed to the target pesticides (as the potentially main source of microbial pesticide-degrading communities; Sniegowski et al., 2011).

The three components of the biomixture are usually mixed at a volumetric proportion of 2:1:1; such composition has not been properly defined with scientific rigor, instead it corresponds to a traditionally employed proportion, commonly applied at on-farm level in some regions (Karanasios et al., 2012). In this respect, recent works aiming at improving the removal performance of biomixtures have revealed optimized compositions that significantly differ from the conventional 2:1:1 proportion (Chin-Pampillo et al., 2015; Ruiz-Hidalgo et al., 2016). Consequently, the need to optimize the biomixture composition for each case has been highlighted, depending on the availability of materials employed in its preparation and the target pesticide(s) (Acosta-Sánchez et al., 2020), mostly considering the specific combinations of pesticides applied on a particular region and crop. Furthermore, the assessment of the removal performance of biomixtures towards relevant mixtures of pesticides (those simultaneously applied or at least employed in the same crop) is necessary to enhance their application scope at real farm scale.

This work aims at designing and optimizing a BPS-biomixture for the simultaneous treatment of several pesticides applied in coffee crops. Three biomixtures containing either coffee husk, coconut fiber or rice husk as the lignocellulosic substrate were considered for the removal of 12 pesticides; they were selected considering the diversity of target pests (three insecticides, eight fungicides and one herbicide) and chemical groups (six different groups; see Table 1), as well as their local approved use in coffee fields. The performance of biomixtures regarding the parameters of pesticide elimination, mineralization of radiolabeled chlorpyrifos (^14^C-chlorpyrifos) and detoxification (according to immobilization in *Daphnia magna* and germination tests in *Lactuca sativa*) were employed to select the biomixture of best performance, whose composition was subsequently optimized by using a central composite design (CCD) considering the volumetric content of the components as design variables. After validation of the optimized composition, and considering the removal kinetics, a 1 m^3^ prototype was dimensioned to be eventually used in local coffee farms. The findings from this work provide useful input for the enhancement and implementation of BPS at farm conditions.

**Table 1:** Pesticides (active ingredients, a.i.) employed in this work.

Pesticide (a.i.)	Commercial formulation	Concentration in the commercial formulation	Chemical group
Oxamyl	Oxate 24 SL	240 g L^−1^	Carbamate
Thiophanate-methyl	Cycosin 50 SC	500 g L^−1^	Benzimidazole
Hexaconazole	Hexil 5 SC	50 g L^−1^	Triazole
Fluazifop-p-butyl	Flob 12.5 EC	125 g L^−1^	Aryloxyphenoxypropionate
Carbendazim	Soprano 25 SC	250 g L^−1^	Benzimidazole
Epoxiconazole	Soprano 25 SC	250 g L^−1^	Triazole
Propiconazole	Propicon 25 EC	250 g L^−1^	Triazole
Triadimefon	Next 25 WP	250 g kg^−1^	Triazole
Chlorpyrifos	Solver 48 EC	480 g L^−1^	Organophosphate
Imidacloprid	Armero 70 WG	700 g kg^−1^	Neonicotinoid
Tebuconazole	Silvacur Combi 30 EC	225 g L^−1^	Triazole
Triadimenol	Silvacur Combi 30 EC	75 g L^−1^	Triazole

## 2 Materials and methods

### 2.1 Pesticide commercial formulations, standards and chemicals

A selection of commercial formulations of insecticides and fungicides approved by the Costa Rican Servicio Fitosanitario del Estado for use in coffee crops were purchased from local markets; the commercial formulations and details regarding the respective active ingredients are enlisted in Table 1. Analytical standards oxamyl, thiophanate-methyl, hexaconazole, fluazifop-p-butyl, carbendazim, epoxiconazole, propiconazole, triadimefon, chlorpyrifos, imidacloprid, tebuconazole and triadimenol were obtained from Chem Service Inc. (West Chester, Pennsylvania, United States). Radio-labeled chlorpyrifos ([ring-2,6-^14^C_2_]-chlorpyrifos; 4.38 × 10^9^ Bq g^−1^; radiochemical purity 98.99%; chemical purity 98.34%) was obtained from Izotop (Institute of Isotopes Co., Budapest, Hungary). Carbofuran-d_3_ (surrogate standard, 99.5%) and linuron-d_6_ (internal standard, 98.5%) were purchased from Dr. Ehrenstorfer (Augsburg, Germany). Potassium hydroxide and sodium hydroxide (analytical grade) were purchased from Merck (Darmstadt, Germany). Ultima Gold cocktail for liquid scintillation counting was purchased from Perkin Elmer (Waltham, Massachusetts, United States). Solvents and extraction chemicals are listed in Ruiz-Hidalgo et al. (2014).

### 2.2 Biomixture components and preparation

Soil was collected from the upper soil layer (0–20 cm) of two coffee fields, located in Desamparados (S1) and Palmares (S2), Costa Rica, and sieved through a 2 mm sieve. Garden compost, employed as the humic component, was purchased from a local market. Coconut fiber (CF; from a local market), rice husk (RH; from a local market) and coffee husk (CH; from a coffee farm in Palmares, Costa Rica) were used as lignocellulosic substrates.

**Figure 1:**
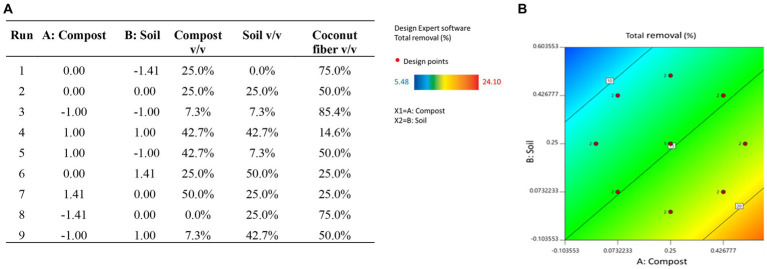
Biomixture compositions evaluated during the optimization using a Central Composite Design (CCD) analysis **(A)**. Surface response analysis of the CCD for the variable “removal,” using the biomixture of best performance (coconut fiber) **(B)**.

Three biomixtures, each containing one lignocellulosic substrate, were prepared by mixing the compost, soil (S1) and either CF, RH or CH at the volumetric ratio 1:1:2 (screening phase), to obtain the CF-, RH- and CH-biomixtures. For optimization purposes, biomixtures containing CF, compost and soil (S1) were prepared at the volumetric ratios shown in Figure 1A in order to obtain a total of nine biomixtures of different composition, according to the design variables described in section 2.3. The biomixtures were moistened to approximately 75% of the maximum water-holding capacity and aged at 25°C during one month prior to use in removal assays.

### 2.3 Experimental design, surface response methodology and statistical analysis

A CCD methodology with two factors (*k* = 2) was applied to optimize the composition of the CF-containing biomixture, in terms of the volumetric content of each of the biomixture components, as previously described (Chin-Pampillo et al., 2015). The design variables or factors were the volumetric content of compost (%) (*A*) and the volumetric content of soil S1 (%) (*B*); the third component content (CF in this case) remains a dependent variable. The effect of these factors was determined on the individual and total pesticide removal as response variables. CCD employs 2*^k^* factorial points representing all combinations of the codified values (±1), 2*^k^* axial points at a distance ±α from the origin, and at least three central points in the origin (encoded as 0,0). The factor levels were normalized and coded in the range (−α, +α). The α value corresponds to 1.414 (α = *F*^1/4^, where *F* = 2*^k^*).

Nine combinations of the design variables were evaluated; to determine the experimental uncertainty, the central point was performed by quintuplicate. This resulted in an experimental design that included 13 runs. The experimental design matrix is shown in Figure 1A and includes actual values for the different combinations of factors *A* and *B*. The CCD was centered in the point where *A* = 25% and *B* = 25%, which corresponds to the volumetric composition employed in the screening phase, normally applied in biomixtures. Removal assays were performed as described in section 2.4.1.

Each response variable can be fitted to a second order polynomial model (*k* = 2), according to the Eq. 1:


(1) $$y=b_{0}+b_{1}A+b_{2}B+b_{12}\mathrm{AB}+b_{11}A^{2}+b_{22}B^{2}$$

The estimation of the model parameters (*b*_i_) and the statistical analysis were performed using the software Design Expert 11 (Stat-Ease Inc., Minneapolis, United States). The quality of the fit polynomial model was determined by the Fisher’s *F*-test; model terms were evaluated by the *p*-value with 95% confidence level; and results were completely analyzed by analysis of variance (ANOVA) employing the same software. Optimization of the biomixture composition was conducted with surface response methodology (SRM) by the analysis of contour plots and numerical solutions by the software, to maximize pesticide removal after 30 d. Besides the optimization based on equal weight assigned to the removal of each pesticide, optimization was also done considering other criteria to assign different weight to the pesticides: i. maximum removal; ii. persistency; iii. Acute ecotoxicity; iv. human toxicity. The weight assigned for each criterium is shown in Supplementary Table S1.

Two optimal composition trends were determined according to the optimization procedure. Validation removal assays (as described in section 2.4.1) were additionally performed using both compositions; a second biomixture was prepared using soil S2 (from another coffee field) at the optimized compositions to estimate potential variations due to the origin of the soil.

### 2.4 Experimental procedures

#### 2.4.1 Pesticide removal experiments

The simultaneous removal of the mixture of pesticides was assayed in 50 mL polypropylene tubes containing 5 g of biomixture. Each tube was spiked with the commercial formulations (30 mg kg^−1^ each, nominal concentration), manually homogenized and incubated in the dark at (25 ± 1) °C; for each biomixture (screening phase), triplicate tubes were withdrawn for analysis at each sampling point (0, 2, 6, 9, 15, 21, and 30 d) for pesticide quantification. During the optimization phase an analogous procedure was followed, using only the CF containing biomixture at the compositions indicated in Figure 1A; duplicate tubes were prepared per composition, except for the composition corresponding to the central point of the CCD (five replicates); pesticide concentrations were determined at time zero and after a treatment period of 30 d. Additional unitary systems containing 300 g biomixture were sampled at times 0, 15 and 30 d to perform ecotoxicological analysis with *D. magna* and *Lactuca sativa* in the screening phase.

#### 2.4.2 Mineralization of ^14^C-chlorpyrifos

The mineralization of ^14^C-chlorpyrifos was determined through ^14^CO_2_ production in biometric flasks, as described in Huete-Soto et al. (2017a). Briefly, 50 g of each biomixture were spiked (triplicates) with either commercial chlorpyrifos (30 mg kg^−1^) or the mixture of pesticides (30 mg kg^−1^ each), plus ^14^C-chlorpyrifos (5,000 dpm g^−1^) and incubated at 25°C; triplicate blanks without pesticides were included for each biomixture. The KOH (0.25 M) contained in the CO_2_ traps was withdrawn at selected times and replaced with the same amount of fresh KOH during a period of 44–46 d. Activity of ^14^C in the ^14^CO_2_ produced from the mineralized pesticide was analyzed in the KOH samples as described in section 2.5.2.

#### 2.4.3 Microbial respiration of biomixtures

Microbial respiration was determined in the biometric flasks (section 2.4.2), using 10 g biomixture with no addition of pesticides; NaOH (0.55083 M) was added in the CO_2_ trap. Analysis is described in section 2.5.3.

### 2.5 Analytical procedures

#### 2.5.1 Extraction and analysis of pesticides

Extraction of pesticides was carried out following a method described by Ruiz-Hidalgo et al. (2014), which employs a mixture of water and acidified acetonitrile (formic acid 1% v/v) as the extractant. Carbofuran-d_3_ and linuron-d_6_ were added as surrogate and internal standard, respectively. Analysis of sample extracts was performed by LC–MS/MS using ultra high performance liquid chromatography (UPLC-1290 Infinity LC, Agilent Technologies, CA) coupled to a triple quadrupole mass spectrometer (model 6,460). The chromatographic separation method is described in Chin-Pampillo et al. (2015). Selected transitions, limit of detection (LOD) and limit of quantification (LOQ) for the analytes are shown in Supplementary Table S2. Remaining pesticide concentrations were used to calculate the percentage of pesticide removal. Removal rate constants and pesticide half-lives (DT_50_) were estimated in Python 3.9 using either a three parameter (Eq. 2) or a simple modified (Eq. 3) first order exponential decay model:


(2) $$y=y_{0}+ae^{-bt}$$

(3) $$y=ae^{\frac{b}{t+c}}$$

Where *y* is the pesticide concentration, *t* is time elapsed, *y_0_*, *a,b,c* are parameters of the model. Removal rate constants were analyzed by means of ANOVA tests to compare regression lines using the InfoStat.

#### 2.5.2 Determination of ^14^C-CO_2_ from mineralization of ^14^C-chlorpyrifos

Scintillant liquid (10 mL) was added to 2 mL aliquots from the removed KOH solution samples and the ^14^C activity from the trapped ^14^CO_2_ was measured by liquid scintillation (Beckman LS6000SC counter). Mineralization was estimated by comparing the total cumulative ^14^CO_2_ activity evolved from the biomixture and the activity of the ^14^C-chlorpyrifos initially added; data was modeled in Python 3.9.

#### 2.5.3 Determination of CO_2_ from respiration experiments

The CO_2_ captured as Na_2_CO_3_ was precipitated with BaCl_2_, and the remaining NaOH was titrated with HCl; basal respiration was calculated as described in Pell et al. (2006).

#### 2.5.4 Ecotoxicological analysis

Elutriates obtained from biomixture samples were prepared according to the protocol EPA-823-B-01-002 (US EPA, 2001), by adding 40 mL distilled water to 10 g samples followed by mechanical shaking (1 h) and centrifugation (10 min at 3500 rpm). The immobilization test in *D. magna* was performed as described by Lizano-Fallas et al. (2017). Briefly, ten daphnid neonates (triplicates) were exposed to 10 mL of elutriate dilutions in moderately hard reconstituted water (21 ± 1°C for 48 h); immobility of neonates was determined and assumed as equivalent to mortality. The relative concentration of the sample that resulted in 50% of immobilization in the daphnids (EC_50_) was calculated using Python 3.9.

The phytotoxicity of the samples was determined with seed germination tests in lettuce (*Lactuca sativa* var. Georgia) (US EPA, 1996). Relative seed germination (SG), relative root elongation (RE) and germination index (GI) were determined using 10 seeds exposed to elutriate samples (5 mL), after 6 d of incubation in darkness at 22°C. These parameters were determined by comparison to germination controls obtained by exposure to distilled water and were calculated as described elsewhere (Lizano-Fallas et al., 2017).

### 2.6 Statistical analysis

Differences in mineralization and removal data, as well as in ecotoxicological parameters were assessed with ANOVA using the Tukey test (α = 0.05) in InfoStat. The CCD and the respective SRM were analyzed using Design Expert 11 (section 2.3).

## 3 Results and discussion

### 3.1 Biomixture performance: screening of different lignocellulosic substrates

#### 3.1.1 Respiration and mineralization of ^14^C-chlorpyrifos

Respiration of biomixtures was assessed as a preliminary indicator of potential pesticide removal capacity. Respiration data revealed significantly higher initial rates in the CH-biomixture (10.2 ±0.2 mgCO_2_ kg^−1^·h^−1^), compared to RH- (3.83 ± 0.34 mgCO_2_ kg^−1^·h^−1^) and CF-biomixtures (1.47 ± 0.34 mgCO_2_ kg^−1^·h^−1^). Respiration rate is usually considered as a marker of biological activity, which correlates with the capacity of soils to transform organic pollutants (Pascual et al., 2000). Nonetheless, in biomixtures this parameter has been previously determined to estimate the effect of antibiotics of agricultural use on these matrices (Castillo-González et al., 2017; Huete-Soto et al., 2017a).

**Figure 2:**
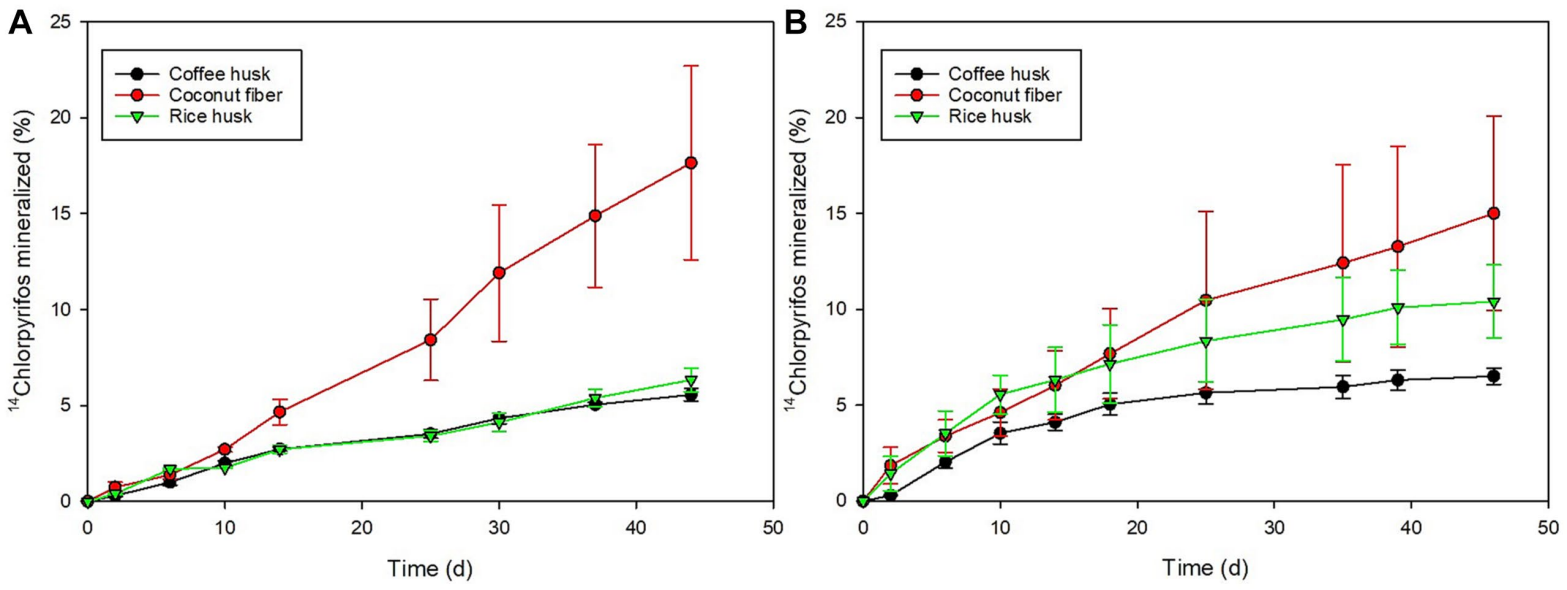
Mineralization of ^14^C-chlorpyrifos represented as % ^14^CO_2_ evolved from the biomixtures over a period of 44–46 days. Biomixtures containing the mixture of 12 pesticide formulations **(A)**; biomixtures containing only the chlorpyrifos formulation **(B)**. Biomixtures: coffee husk (black circles), coconut fiber (red circles), and rice husk (green inverted triangles).

The mineralization of ^14^C-chlorpyrifos was determined as another indicator of pesticide transformation capacity of the matrix. Significantly higher final mineralization values (after 45 d) were achieved in the CF-biomixture in both cases (17.7 ± 6.2% and 15.0 ± 6.2% for chlorpyrifos alone or in the mixture containing the 12 target pesticides, respectively), compared to the other biomixtures (up to 6.5% in the CH-biomixture and 11.0% in the RH-biomixture) (Figure 2). Other studies have reported comparable mineralization values in analogous biomixtures, ranging from 10 to 17.6% after 62 d for ^14^C-chlorpyrifos (Castillo-González et al., 2017; Huete-Soto et al., 2017a) and 13% after 28 d for ^14^C-carbofuran (Jiménez-Gamboa et al., 2018). The mineralization capacity, however, did not correlate with the respiration rates, and conversely, the highest mineralization was observed in the biomixture with the lowest respiration rate. Consequently, parameters directly related to pesticide removal/transformation should be prioritized over matrix respiration when evaluating the performance biomixtures.

#### 3.1.2 Removal of pesticides

**Figure 3:**
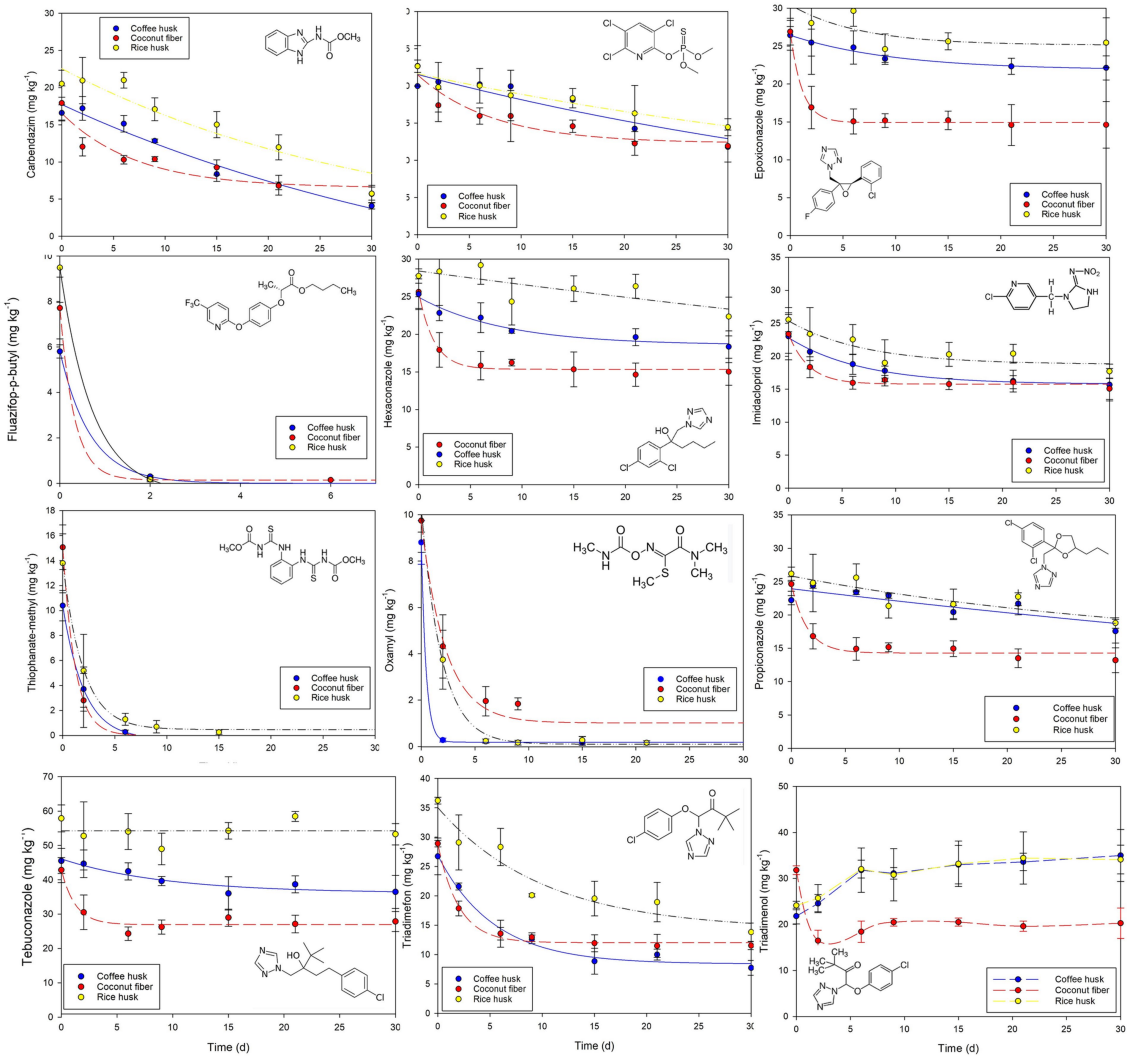
Removal profiles of a mixture of 12 pesticide formulations in biomixtures containing either coffee husk, coconut fiber or rice husk as the lignocellulosic substrate, during a period of 30 d. Values plotted are means ± SD for triplicate systems.

The removal profiles for individual pesticides during the treatment in three different biomixtures are shown in Figure 3. Overall, the CF-biomixture exhibited the best removal performance, achieving a significantly lower final total concentration of pesticides after 30 d of treatment, followed in descendant order by the CH- and RH-biomixtures. Estimated DT_50_ values for individual pesticides are shown in Table 2; given that DT_50_ concentrations were experimentally reached only for five pesticides, no DT_50_ values were estimated in the other cases; instead, removal values (%) by the end of the experiment are reported. Although the elemental composition of the biomixtures was not determined, the differences among their C/N ratios are exclusively related to the lignocellulosic substrate (as the proportion and origin of soil and compost is the same). Given that the C/N for CF (~83) is much higher than those for RH (~23) and CH (~30) (Shemekite et al., 2014; AboDalam et al., 2022), the CF-biomixture has a higher C/N value. The biomixtures with high C/N ratios are known to favor the activity of white rot fungi and their enzymatic machinery, which may enhance the oxidation of organic micropollutants (Yang et al., 2013); this observation might at least partially explain the best pesticide removal observed in this matrix.

**Table 2:** Estimated half-lives (DT_50_) during the simultaneous removal of 12 pesticides using three different biomixtures. Comparison with DT_50_ values in soil and other biomixtures are included.

Pesticide	DT_50_ (d); current study^a^			Soil elimination^c^			Reported removal in biomixtures		Reference
	RH	CH	CF	Typical DT_50_ (d)	Laboratory (20°C) DT_50_ (d)	Field DT_50_ (d)	Biomixture (composition; v/v)	DT_50_ (d)	
Carbendazim	20.7	16.5	12.6	40	34.3	22	Millet stubble–Soil (1:1)	6.8	Lescano et al. (2022)
Chlorpyrifos	NC (36.1)^b^	NC (40.9)	NC (47.4)	386	386	27.6	Coconut fiber–compost – Soil (2:1:1)	56	Masís-Mora et al. (2019)
Epoxiconazole	NC (17.1)	NC (19.4)	NC (45.7)	354	226	120	Soil–compost–wheat straw (1:1:2)	61	Fogg et al. (2003)
Fluazifop-p-butyl	0.3	0.3	0.3	1	3.4	8.2	Wheat straw–*Sphagnum* (peat moss)–Soil (2:1:1)	NR	Spliid et al. (2006)
Hexaconazole	NC (19.5)	NC (27.6)	NC (41.4)	191	187	174	Coconut fiber–compost–Soil (2:1:1)	187.3	Masís-Mora et al. (2019)
Imidacloprid	NC (30.7)	NC (31.9)	NC (35.6)	191	187	174	Millet stubble–Soil (1:1)	83	Lescano et al. (2022)
Tiophanate-methyl	1.44	1.39	0.85	0.5	0.5	2	Rice straw–compost–Soil (2:1:1)	28.9	El-Sebai et al. (2023)
Oxamyl	1.43	0.3	2.32	5.3	5.3	6	Soil and manure in open field	3.19	Osman et al. (2009)
Propiconazole	NC (28.2)	NC (20.9)	NC (46.4)	71.8	71	35.2	Wheat straw–moss–Soil (2:1:1)	160	Spliid et al. (2006)
Tebuconazole	NC (7.9)	NC (19.8)	NC (35.0)	63	365	47.1	Soil–vermicompost–tomato waste (1:2:1)	19	Delgado-Moreno et al. (2017)
Triadimefon	18.3	7.2	4.21	26	NR^d^	NR	NR	–	–
Triadimenol	−4.59	−3.91	NC (36.3)	250	136.7	64.9	Coconut fiber–compost–Soil (2:1:1)	135.9	Masís-Mora et al. (2019)

a Biomixtures: RH = rice husk; CH = coffee husk; CF = coconut fiber.

b NC = not calculated (as the half-life was not experimentally reached); in these cases, the removal (%) by the end of the assay (30 d) is reported in parenthesis.

c According to Lewis et al. (2016).

d NR = not reported or not available in scientific literature.

Half of the evaluated pesticides belonged to the group of triazole fungicides. For these compounds (epoxiconazole, hexaconazole, propiconazole, tebuconazole and triadimefon), most of the removal took place during the first 3–4 days of treatment. However, the removal rate decreased by day five, reaching constant values in most cases, which ultimately did not permit the estimation of DT_50_ values, except in the case of triadimefon whose DT_50_ values (lowest DT_50_ of 4.2 d in CF-biomixture) were below the reported elimination in soil (DT_50_ 26 d; Lewis et al., 2016). In general, triazoles are usually considered as persistent compounds, and their removal in soil ranges from 35 d to 365 d for the remaining compounds employed in this study (Lewis et al., 2016). On the other hand, triazole removal in biomixtures has revealed contrasting results, i.e., from DT_50_ = 19 d (Delgado-Moreno et al., 2017) to negligible elimination for tebuconazole (Murillo-Zamora et al., 2017), or cases of faster (epoxiconazole, DT_50_ = 61 d; Fogg et al., 2003), similar (hexaconazole, DT_50_ = 187 d); or slower (propiconazole, DT_50_ = 160 d; Spliid et al., 2006) elimination compared to reports in soil. It should also be considered that removal behavior might change depending on the combination of pesticides simultaneously occurring in a matrix (Huete-Soto et al., 2017b), likely due to the ecotoxicological effects of the pesticide mixture (and their metabolites) on the potentially degrading microbial communities (Bose et al., 2021).

The concentration profile for triadimenol differs from the other triazoles, as accumulation was observed in the CH- and RH-biomixtures, while initial removal followed by accumulation was determined in the CF-biomixture. Such behavior can be explained as triadimenol is the main transformation product of triadimefon in soil (Hou et al., 2022). Hence, data suggest that faster initial removal of triadimenol in the CF-biomixture was then masked by the transformation of triadimefon (and the consequently production of triadimenol); on the contrary, the accumulation rate of newly produced triadimenol was always higher than triadimenol removal rates in the other biomixtures, thus resulting in its net accumulation. Longer treatment periods are recommended to estimate the extent of triadimenol removal in the biomixtures, more importantly considering that its DT_50_ in soil is around ten-times longer than that of triadimefon (26 d versus 250 d; see Table 2) (Hou et al., 2022).

Three compounds (fluazifop-p-butyl, oxamyl, thiophanate-methyl) showed almost complete removal after short treatment periods. Fluazifop-p-butyl showed shorter DT_50_ values (estimated DT_50_ = 0.3 d in all biomixtures) compared to its removal in soil (Lewis et al., 2016). In the case of oxamyl the removal rates (DT_50_ ranging from 0.3 d in the CH-biomixture to 2.32 in the CF- biomixture) were higher than previously reported in soil and biomixtures (Osman et al., 2009; Lewis et al., 2016). Similarly, the dissipation rate for thiophanate-methyl (DT_50_ ranging from 0.85 d in the CF-biomixture to 1.44 d in the RH-biomixture) was within the removal values in soil (Lewis et al., 2016), but shorter than reported by El-Sebai et al. (2023) in a rice husk-compost-based biomixture.

The removal of carbendazim, although not complete, exhibited DT_50_ values ranging from 12.6 d (CF-biomixture) to 20.7 d (RH-biomixture); they were in general faster than observed dissipation rates in soil, but similar or longer than estimated in other biomixtures (Castillo-González et al., 2017; Huete-Soto et al., 2017b; Lescano et al., 2022). Moderate removal was observed for the organophosphate chlorpyrifos (maximum of 47.4% after 30 d in the CF-biomixture), whose elimination in biomixtures has shown DT_50_ values ranging from 9 d to 69 d (Kravvariti et al., 2010; Masís-Mora et al., 2019; Pérez-Villanueva et al., 2022). The described accumulation of the chlorpyrifos metabolite 3,5,6-trichloropyridinol (TCP) in biomixtures (Tortella et al., 2012) is known to potentially delay chlorpyrifos degradation due to the toxic effect TCP exerts on degrading microbial communities (Bose et al., 2021). Finally, imidacloprid is highly persistent both in soil (Lewis et al., 2016) and biomixtures; reports in the latter indicate a DT_50_ value of 83 d (Lescano et al., 2022) or even negligible dissipation (Huete-Soto et al., 2017b). Although some extent of removal was achieved in this work (up to 35.6% after 30 d in the CF-biomixture), imidacloprid dissipation reached a more or less constant value after 15 d. Significantly longer treatment periods are likely to be required to remove such highly persistent compounds, or the use of more than one BPS at on-farm level, to treat more recalcitrant compounds (Acosta-Sánchez et al., 2020).

#### 3.1.3 Ecotoxicological changes during pesticide removal

**Figure 4:**
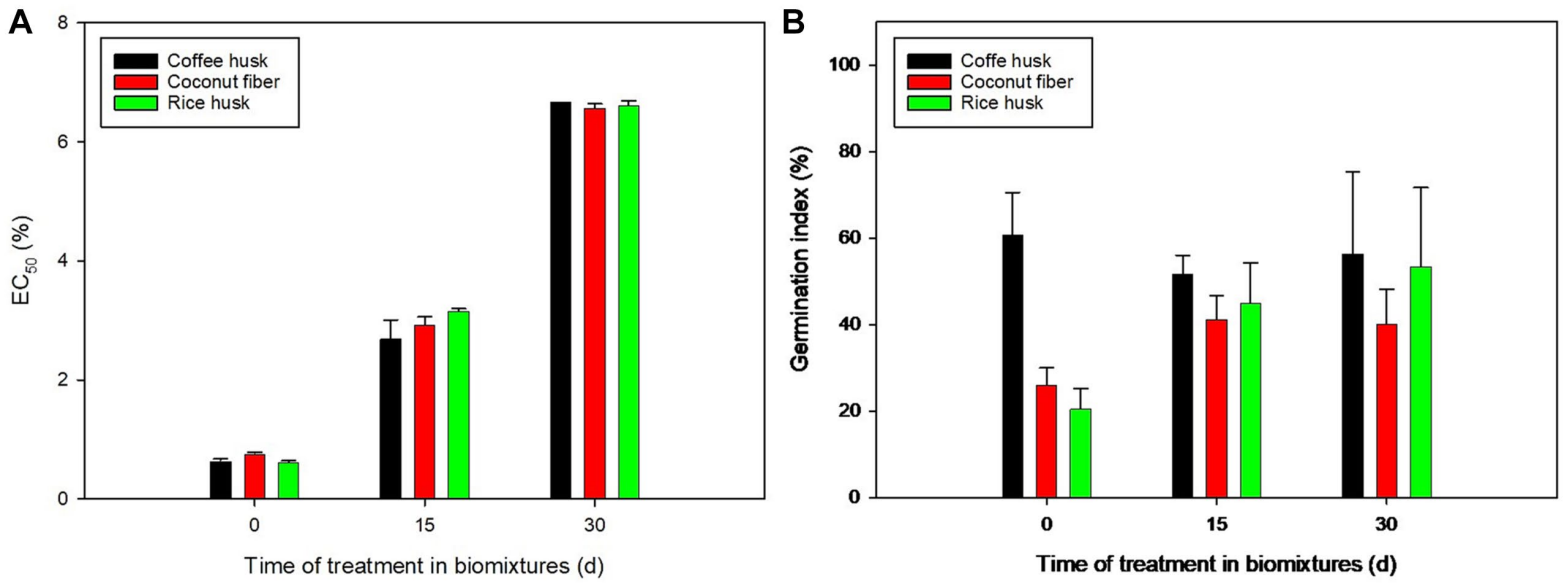
Variations in the ecotoxicity of the biomixtures during the removal of a mixture of 12 pesticides. Ecotoxicological tests: immobilization of *D. magna*, reported as EC_50_ **(A)**; germination assays in *Lactuca sativa*, reported as germination index **(B)**.

Ecotoxicological assays were performed in *D. magna* and *Lactuca sativa* to monitor the removal of pesticides in the biomixtures. The immobilization test in *D. magna* (Figure 4A) showed a steady significant detoxification in the biomixtures during the treatment period, shifting from initial EC_50_ values ranging from 0.65–0.77% to 2.70–3.17% after 15 d and to 6.57–6.67% after 30 d, in a detoxification pattern that did not exhibit significant differences among the three biomixtures. Despite differences in the pesticide removal performance of biomixtures, the similarity in their ecotoxicological outcome supports the versatility of these matrices, in which detoxification may be also related to adsorption processes during the aging of the biomixtures (Castillo et al., 2008). Nevertheless, the detoxification due to transformation of the parent pesticides remains the most desired process from the bioremediation point of view, as long as toxic transformation products are not formed or accumulated. Similar detoxification towards *D. magna* was also observed in compost-based biomixtures used for the treatment of seven fungicides (including carbendazim, epoxiconazole, tebuconazole, and triadimenol) (Murillo-Zamora et al., 2017), a mixture of triazines plus chlorpyrifos (Lizano-Fallas et al., 2017) or mixtures of carbamates (Rodríguez-Rodríguez et al., 2017). On the contrary, fluctuations in the toxicity towards *D. magna* with no net detoxification were described during the treatment (115 d) of mixtures of up to nine pesticides (including four used in this work) (Huete-Soto et al., 2017b); moreover, increased toxicity has been reported, which may take place during the formation of highly toxic transformation products or due to the accumulation of specific pesticides in cases of several applications on the biomixture (Acosta-Sánchez et al., 2020).

Among the treated pesticides, chlorpyrifos is by far the most toxic to *D. magna* (EC_50_ = 0.00010 mg L^−1^; Lewis et al., 2016), and is likely the most important compound within the mixture to shape the final ecotoxicological outcome in the matrix, while carbendazim (EC_50_ = 0.15 mg L^−1^) and oxamyl (EC_50_ = 0.319 mg L^−1^) follow in toxicity. All these three compounds were significantly removed in the biomixtures, which might explain the overall decrease in their residual toxicity. Nonetheless, the final toxicity will depend on the interactions between the complex mixture of pesticides and their transformation products (Hernández et al., 2017). The adsorption of the pesticides during the aging of the matrix might also decrease their bioavailability (and their biological removal) (Castillo et al., 2008), which may contribute to the observed detoxification. Even though detoxification was achieved in this work, longer treatments are recommended to further decrease the residual toxicity.

Germination tests in *Lactuca sativa* showed a similar trend to that observed in daphnids: a significant detoxification in the CF- and RH-biomixtures after 30 d of treatment, with respect to the initial toxicity (Figure 4B). No detoxification was observed in the CH-biomixture, as the initial GI value (higher compared to the other matrices) did not differ from the GI at times 15 d or 30 d; such unexpected high initial GI could be related to specific pesticide adsorption to the CH-biomixture, that hindered pesticide release during the aqueous extraction. The decrease in the toxicity towards *Lactuca sativa* has been described during the removal of herbicides in biomixtures (Acosta-Sánchez et al., 2020) and during the treatment of pesticide mixtures (Huete-Soto et al., 2017b). As in the case of the test in *D. magna*, final toxicity values were not significantly different regardless of the biomixture. For this reason, although detoxification was achieved, the ecotoxicological data did not play a key role in defining the biomixture of best performance for further optimization assays.

### 3.2 Optimization of biomixture composition

Due to its better mineralization and removal performance, the CF-biomixture was selected for further optimization of the composition of the matrix. As indicated above, the ecotoxicological results did not permit the use of this parameter as a selection criterion.

Considering the overall removal profiles obtained in the screening phase, a treatment time of 30 d was selected to calculate the removal of the pesticides (response variables) in each of the biomixture compositions defined in Figure 1A according to the CCD. Such removal values after 30 d are shown in Supplementary Table S3. The SRM was employed for the correlation analysis between the design variables (compost content % v/v; soil content % v/v) and pesticide removal, and to determine the optimized composition of the biomixture. “Flat” SRM analysis (same weight of every factor [pesticide] on the assay) was complemented with a “weighted” SRM analysis, in which different weights were assigned to each factor or pesticide, according to the following criteria: i. maximum removal (according to results in section 3.1); ii. pesticide persistence (PPDB: pesticide database; Lewis et al., 2016); iii. Acute human toxicity;^1^ and iv. Acute ecotoxicity (PPDB: pesticide database; Lewis et al., 2016). Optimization based on removal maximization (taking into account the assigned weights) revealed two optimal conditions in the contour plots (see Figure 1B), thus resulting in the following optimized biomixture compositions (compost:soil:CF, % v/v): i. 29:7.3:63.7 and ii. 11:7.3:81.7. Interestingly, both compositions differ from the traditional 1:1:2 compositions, mostly on the decreased content of soil and the increased content of the lignocellulosic substrate. This result correlates the findings by Sniegowski et al. (2012), who suggest that low content of primed-soil of around 0.5% (v/v) is sufficient to supply the microbial degrading communities required for the operation of a biomixture. A similar optimization approach employed in biomixtures aimed for the specific removal of the carbamate carbofuran also showed important deviations with respect to the traditional composition, either by the increase in soil content (13:42:45, compost:soil:CF, % v/v; Chin-Pampillo et al., 2015) or the increase in compost content in a bioagumented matrix (43:27:30, compost:soil: CF, % v/v; Ruiz-Hidalgo et al., 2016).

For the validation of the optimization process, biomixtures with the respective optimal compositions were prepared (OP29: optimal composition 29:7.3:63.7; OP11 optimal composition 11:7.3:81.7) and employed for the removal of the pesticide mixture during 20 d. In this case, two different soils were used for biomixture preparation: the original soil so far employed in screening and optimization phases (S1), and a second soil from another coffee farm (S2), to obtain four biomixtures (OP29-S1, OP29-S2, OP11-S1, OP11-S2). Comparison of half-lives for the non-optimized (section 3.1) and these optimized biomixtures are shown in Table 3. As observed in the non-optimized biomixtures, the removal sharply decreased in the optimized biomixtures after 5 d of treatment; again, half-life values were not experimentally reached for triazoles and imidacloprid. This finding might suggest the potential need to use a second optimized biomixture (and probably longer treatment periods) for these persistent compounds (Acosta-Sánchez et al., 2020). Similarly, the combined toxicity of the pesticide mixture applied in the matrix may cause more inhibition on the microbial degrading activity; hence, the use of separate biomixtures could further decrease this toxic burden. Remarkably, the removal of chlorpyrifos, the most toxic compound towards *D. magna*, was improved in the optimized biomixtures, reaching DT_50_ values ranging from 8.6 d to 17.8 d, lower than typically reported in these matrices (Kravvariti et al., 2010; Masís-Mora et al., 2019; Pérez-Villanueva et al., 2022). Similarly, the elimination of carbendazim was significantly enhanced, going from DT_50_ = 16.5 d in the non-optimized matrix, to values from 1.9 d to 7.5 d in the optimized biomixture. DT_50_ values for fluazifop-p-butyl and thiophanate-methyl were similar in optimized and non-optimized conditions; on the other hand, a slight increase in DT_50_ was only recorded for oxamyl in the optimized matrices, however the values are still low (< 2.2 d) compared to soil or other biomixtures (Osman et al., 2009 Lewis et al., 2016). Contrary to observed in the non-optimized matrix, the global half-life for the pesticide mixture was reached and estimated in the biomixtures OP11-S1 (DT_50_ = 9.0 d) and OP11-S2 (DT_50_ = 7.8 d). Overall, the analogous removal profiles achieved in both optimized biomixtures (non-significantly different for total pesticide concentration) revealed the success of using an alternative coffee-farm soil, thus suggesting the possibility of employing local soil (at least in this tropical region) for the production of biomixtures.

**Table 3:** Estimated half-lives (DT_50_, days) during the simultaneous removal of 12 pesticides using non-optimized and optimized biomixtures containing coconut fiber (CF) as the lignocellulosic substrate.

	Non-optimized biomixture	Optimized biomixtures			
Pesticide		29:7.3:63.7^a^		11:7.3:81.7^a^	
	1: 1: 2^a^	OP29-S1	OP29-S2	OP11-S1	OP11-S2
Carbendazim	16.46^b^	7.51	NC	4.61	1.86
Chlorpyrifos	NC	13.07	17.81	8.55	14.58
Epoxiconazole	NC	NC	NC	NC	NC
Fluazifob-P-butyl	0.30	0.84	0.78	1.31	1.56
Hexaconazole	NC	NC	NC	NC	NC
Imidacloprid	NC	NC	NC	NC	NC
Thiophanate-methyl	1.39	1.06	0.97	1.8	1.57
Oxamyl	0.3	2.15	1.30	1.92	2.16
Propiconazole	NC	NC	NC	NC	NC
Tebuconazole	NC	NC	NC	NC	NC
Triadimefon	7.20	NC	NC	NC	NC
Triadimenol	−3.91	NC	NC	NC	NC
**Global DT_50_**	**NC**	**NC**	**NC**	**8.99**	**7.83**

^a^Volumetric composition (compost:soil:CF).

^b^NC = not calculated (half-life was not experimentally reached).

### 3.3 Design of a biopurification system

The dimensional design of the BPS was done considering the three parameter first order exponential decay model (Eq. 2), and a hydraulic residence time (HRT) equal to the DT_50_ for the sum of pesticides (Eq. 4)


(4) $$HRT=DT_{50}=\frac{V}{Q}$$

where *V* is the minimum biomixture volume required for the BPS, and *Q* is the estimated pesticide-containing wastewater produced on-farm (during the triple washing of equipment and formulation bottles) in a specific period (flow). In the case t = HRT = DT_50_, and considering a BPS of rectangular base, Eq. 2 can be rewritten as as Eq. 5:


(5) $$y_{DT_{50}}=y_{0}+ae^{-b\left[\frac{\left(l\cdot w\right)\;h}{Q}\right]}$$

Where *l* = length, *w* = width, and *h* = height of the BPS. Similarly, from Eq. 2, $y_{DT_{50}}$ can be also expressed as Eq. 6:


(6) $$y_{DT_{50}}=\frac{y_{0}+a}{2}$$

Equating (5) and (6) and solving in terms of the base area of the BPS ( l⋅w ), Eq. 7 is obtained:


(7) $$l\cdot w=\ln\left(\frac{2a}{a-y_{0}}\right)\times \frac{Q}{b\cdot h}$$

Although recommended height ranges from 1–1.5 m, depending on the wastewater load (Fogg et al., 2004), the estimated flow is low in this case and the height was fixed at *h* = 0.80 m; higher values would promote undesirable anoxic environments, mostly at the bottom of the biomixture layer, while lower values may foster leachate production. The flow *Q* depends on the local pesticide application practices in the farm, and the recommended volume for triple rinsing of equipment and bottles. Hence, *Q* was estimated considering the following criteria for local farms: i. pesticide yearly applications range from two to five for coffee crops (wastewater production is not continuous); ii. an average coffee farm area of 3 ha (INEC, 2014); iii. two pesticide application devices (25 L each) per farm; iv. a single rinsing should not exceed 25% of the container capacity. These conditions yield a total of 56.3 L of wastewater produced during each application (including a safety factor), which must be disposed of at the moment; thus, resulting in a design input flow *Q* = 0.54 m^3^/d. Taking into account the cases in which the overall DT_50_ value was obtained in the optimized biomixtures (Table 3), the BPS dimensions were calculated for both scenarios: a base area of 0.59 m^2^ (V = 0.47 m^3^) for OP11-S1, and 0.68 m^2^ (V = 0.54 m^3^) for OP11-S2, and they represent the minimum recommended for the BPS operation.

**Figure 5:**
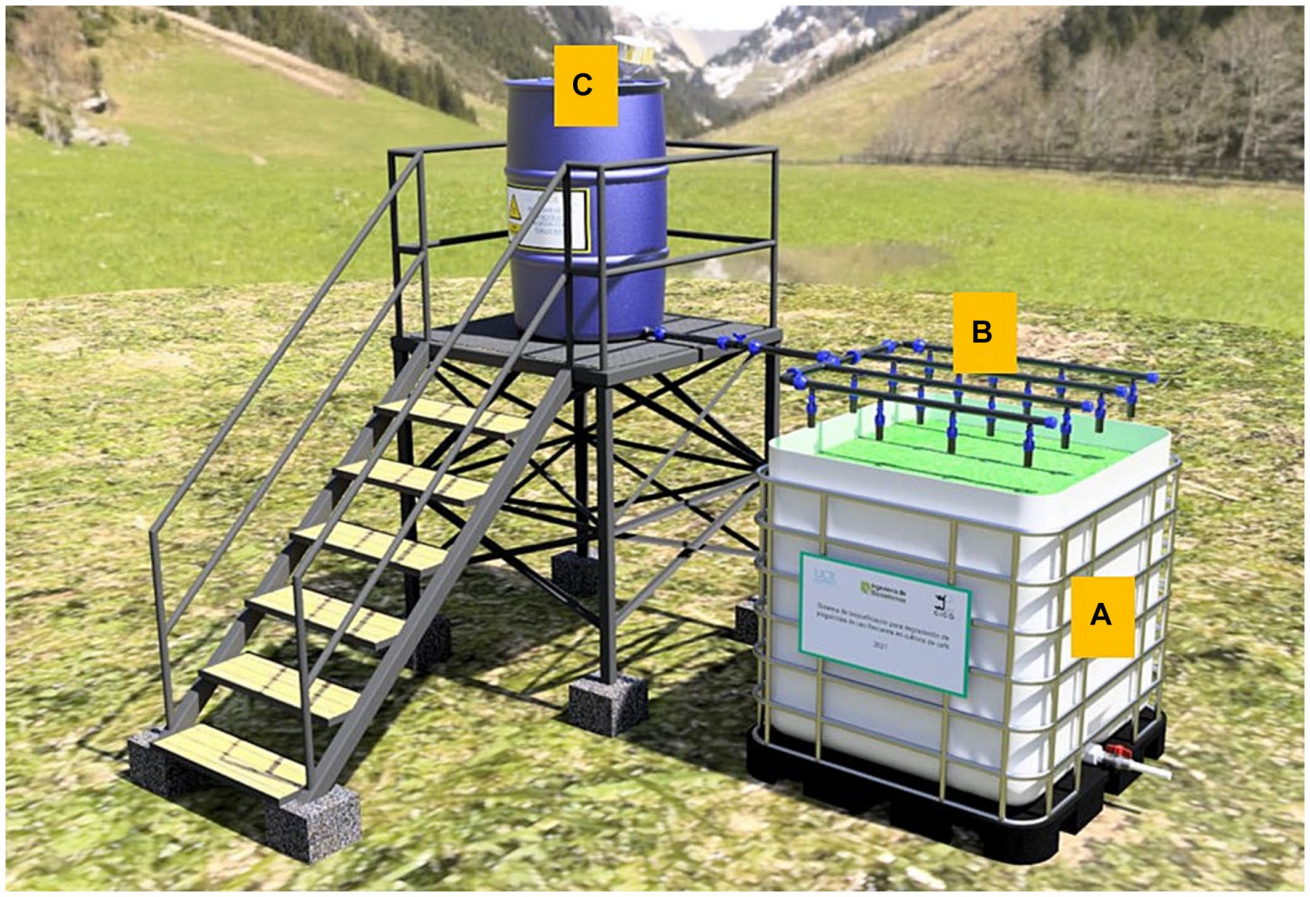
Final design of a 1 m^3^ BPS for the removal of pesticides employed in coffee crops. Subsystems: BPS itself **(A)**; hydraulic system **(B)**; homogenization/reservoir chamber **(C)**.

As the volume of the BPS required for local coffee farms is relatively low, the system was redesigned to make use of cheap available construction materials. Thus, the final design (shown in Figure 5) was composed of the following subsystems:

- The BPS itself: composed of a reused tank (1 × 1 × 1 m), filled with the optimized biomixture at a height of 0.8 m, which is watered by a hydraulic system. A valve at the bottom level of the tank is recommended for purging and collecting leachates, where residual pesticide concentrations are usually reported as low/negligible (Fogg et al., 2004; Kumari et al., 2023).
- Hydraulic system: consisting of an array of pipes with valves working as outlets in parallel to the surface of the BPS; they are placed in a rectangular arrangement of 0.25 × 0.25 m, for a total of 16 outlets that irrigate the biomixture at the defined flow. A main valve allows the wastewater to be loaded into the biomixture.
- Homogenization/reservoir chamber: consisting of a reused 200 L-container, which rests at 0.5 m above the biomixture level. This device acts as a reservoir to dispose of and homogenize the wastewater, before being drained by gravity into the BPS by means of the hydraulic system.

## 4 Conclusion

Analytical, radioisotope and ecotoxicological methods were employed for the selection of a biomixture aimed for the treatment of 12 pesticides used in coffee production. All the evaluated biomixtures were capable of detoxifying to a similar extent the mixture of pesticides; however, the CF-biomixture was the most efficient matrix for the removal of pesticides and the mineralization of ^14^C-chlorpyrifos. The subsequent optimization of this biomixture by means of CCD analysis revealed an optimized composition (11:7.3:81.7, compost:soil: CF) that significantly differs from the typically employed biomixtures. The removal profile in the optimized biomixture permitted the design of a BPS adapted for small local farms, based on the use of mostly low-cost materials. This work remarks the importance of optimizing the biomixture composition to maximize the pesticide removal and the detoxification of the matrix. Although a time-consuming process, optimization should be applied for each specific crop, and for the specific combination of pesticides applied to a single crop. Since a determined biomixture may not be capable of removing every pesticide applied (as suggested by the observed slower removal of triazoles compared to other chemical groups), the use of more than one BPS with specifically design biomixtures, might be a necessary practice to cover the proper treatment of the whole battery of pesticides used in a single farm.

## Data availability statement

The original contributions presented in the study are included in the article/Supplementary material, further inquiries can be directed to the corresponding author.

## Author contributions

FOM: Conceptualization, Formal analysis, Investigation, Methodology, Software, Writing – original draft. MEPV: Methodology, Writing – review & editing. MMM: Investigation, Writing – review & editing. RAÁ: Conceptualization, Writing – review & editing. DRM: Investigation, Writing – review & editing. MMR: Investigation, Writing – review & editing. CERR: Conceptualization, Funding acquisition, Methodology, Project administration, Supervision, Writing – review & editing.

## Footnotes

^1^ http://www.plaguicidasdecentroamerica.una.ac.cr/index.php/base-de-datos-menu
