# Supplementary material for: Biological treatment of pesticide-containing wastewater from coffee crops: selection and optimization of a biomixture and biobed design
Source: Front Microbiol. 2024 Feb 6;15:1357839. doi: 10.3389/fmicb.2024.1357839 (PMC10881177; doi:10.3389/fmicb.2024.1357839)
Supplement: Supplementary file 1 [file Data_Sheet_1.PDF]

Supplementary Material

Biological treatment of pesticide-containing wastewater from coffee crops: selection and optimization of a biomixture and biobed design

Fernando Oviedo-Matamoros [a]; Marta E. Pérez-Villanueva [a]; Mario Masís-Mora [a]; Rónald Aguilar-Álvarez [b]; Didier Ramírez-Morales [a]; Michael Méndez-Rivera [a]; Carlos E. Rodríguez-Rodríguez [a,]*

[a] Centro de Investigación en Contaminación Ambiental (CICA), Universidad de Costa Rica, 2060 San José, Costa Rica.*

[b] School of Biosystems Engineering, Universidad de Costa Rica, 2060 San José, Costa Rica

*Corresponding author: E-mail address, carlos.rodriguezrodriguez@ucr.ac.cr; tel: +506 2511-3719

Table S1. Weight assigned to different criteria during the analysis of the CCD for the optimization of biomixture composition.

Pesticide	Criteria			
	**Maximum removal in screening phase**	**Persistence**	**Acute human toxicity**	**Acute ecotoxicology**
Carbendazim	++++	++++	++++	++
Chlorpyrifos	++	++++	++++	++++
Epoxiconazole	++	+++	++++	+++
Fluazifop-P-butyl	++++	++	++++	+++
Hexaconazole	++	+++	++++	++
Imidacloprid	+++	+++	++++	++++
Tiophanate-methyl	++++	++	++++	++
Oxamyl	++++	++	++++	++++
Propiconazole	++	++++	++++	++++
Tebuconazole	++	++++	++++	+++
Triadimefon	++	++	++++	+++
Triadimenol	NA	NA	NA	NA

NA: Triadimenol was not considered for the analysis, as it is also produced during the transformation of triadimefon

Table S2. Selected transitions and other parameters in the detection of pesticides in the biomixture, using the dynamic multiple reaction monitoring (dMRM) method.

Compound	Transition		Fragmentor (V)	Collision energy (V)	Retention time (min)	Type of transition	LOD (µg kg $^{-1}$)	LOQ (µg kg^{-1})
	Precursor ion	Product ion						
Carbendazim	192	160	94	17	1.55	Q	12	24
		132		33		q		
Chlorpyrifos	350	97	90	30	15.74	Q	37	67
		198		15		q		
Epoxiconazole	330	121	106	25	12.44	Q	23	44
		101		40		q		
Fluazifop-p-butyl	384	282	126	17	14.90	Q	12	24
		328		13		q		
Hexaconazole	314	70	116	21	13.59	Q	14	27
		159		33		q		
Imidacloprid	256	209	72	13	2.14	Q	21	39
		175		17		q		
Thiophanate-methyl	343	151	94	17	7.31	Q	23	46
		301		5		q		
Oxamyl	242	72	106	17	1.253	Q	16	30
		121		9		q		
Propiconazole	342	159	126	29	13.43	Q	18	35
		69		21		q		
Tebuconazole	308	70	106	21	13.25	Q	13	26
		125		40		q		
Triadimefon	294	69	94	21	11.67	Q	9	18
		197		13		q		
Triadimenol	296	70	72	9	12.01	Q	6	13
		99		13		q		
Linuron-d_6 (i.s.)	255	160	92	17	10.62	Q	NA	NA
		185		13		q		
Carbofuran-d_3 (s.s.)	225	165	86	9	7.29	Q	NA	NA
		123		21		q		

Q: quantification transition, q: qualifier transition
i.s.: internal standard; s.s.: surrogate standard

Table S3. Pesticide removal achieved in biomixtures of different composition after a treatment of 30 d. Compositions are defined according to the CCD employed for the optimization of biomixture composition.

Run	Sample	Compost fraction (%)	Soil fraction (%)	Carbendazim	Chlorpyrifos	Epoxiconazole	Fluazifop-p-butyl	Hexaconazole	Imidacloprid	Oxamyl	Propiconazole	Tebuconazole	Thiophanate-methyl	Triadimefon	Triadimenol	Sum of pesticides
1	R1	25%	0%	45,0	3,1	1,9	94,2	2,2	2,8	62,1	1,6	2,1	86,7	4,1	-13,1	18,0
2	R1	25%	0%	45,0	9,7	6,0	93,3	5,3	2,8	62,1	9,0	10,1	86,3	7,9	-11,1	16,3
3	R2	25%	25%	14,3	6,3	4,8	87,9	4,8	10,3	1,6	3,3	3,7	52,2	8,3	-11,3	14,5
4	R2	25%	25%	44,7	10,1	5,4	86,4	6,9	6,3	58,9	9,2	8,5	50,6	10,9	-8,3	5,5
5	R2	25%	25%	9,0	5,3	3,6	87,5	4,3	6,1	37,7	4,4	4,1	48,9	7,7	-11,6	16,6
6	R2	25%	25%	20,5	11,2	9,4	88,2	9,1	11,9	36,6	9,4	10,3	43,3	12,5	-4,1	21,6
7	R2	25%	25%	13,9	2,2	3,5	87,1	3,5	9,5	30,5	1,3	6,7	58,2	6,9	-7,0	15,0
8	R3	7%	7%	14,7	11,0	6,1	93,0	9,4	10,7	28,4	8,1	7,3	59,8	11,7	-14,8	16,9
9	R3	7%	7%	17,9	10,1	8,7	92,9	6,3	11,3	28,4	9,1	8,9	53,1	11,0	-15,9	14,4
10	R4	43%	43%	7,8	13,0	9,8	86,6	10,8	11,9	30,0	11,0	10,0	46,7	12,5	-1,6	18,8
11	R4	43%	43%	6,7	14,4	10,9	87,0	11,5	14,3	21,7	11,0	12,6	33,4	13,3	-1,5	16,7
12	R5	43%	7%	22,8	4,0	3,7	93,8	3,8	3,6	62,0	4,5	4,3	78,4	6,1	-8,5	17,0
13	R5	43%	7%	12,5	12,6	11,4	95,0	10,5	18,4	64,6	10,8	11,1	86,8	11,9	-2,1	24,1
14	R6	25%	50%	38,7	6,1	2,3	76,8	3,0	4,5	73,7	3,7	2,5	6,9	4,7	-9,0	7,8
15	R6	25%	50%	13,3	4,2	2,4	76,6	1,2	4,5	52,7	2,8	4,0	33,1	4,7	-9,0	11,4
16	R7	50%	25%	9,9	8,4	6,8	92,1	7,0	15,0	60,1	5,7	7,4	34,6	8,8	-3,1	15,9
17	R7	50%	25%	10,4	6,3	4,1	90,9	4,1	7,4	67,9	3,4	2,0	26,1	6,3	-4,1	12,8
18	R8	0%	25%	37,5	4,1	3,7	61,0	4,0	10,5	48,7	4,3	3,9	53,8	6,2	-13,0	12,7
19	R8	0%	25%	17,8	4,1	3,7	56,9	4,0	3,0	33,9	4,3	3,9	47,9	0,5	-15,9	8,4
20	R9	7%	43%	12,9	8,5	4,3	65,9	4,0	6,1	40,2	5,0	3,7	47,7	7,1	-9,3	13,8
21	R9	7%	43%	20,5	4,0	3,2	67,2	2,9	7,2	40,2	2,6	0,6	52,8	6,7	-11,2	11,7
